# Is there a benefit of first- or second-line crizotinib in locally advanced or metastatic anaplastic lymphoma kinase-positive non-small cell lung cancer? a meta-analysis

**DOI:** 10.18632/oncotarget.13191

**Published:** 2016-11-07

**Authors:** Hao Hu, Wei Qing Lin, Qian Zhu, Xiong Wen Yang, Hai Dong Wang, Yu Kang Kuang

**Affiliations:** ^1^ Department of Thoracic Surgery, Medical College of Nanchang University, Nanchang, 330006, China; ^2^ Department of Integrated Chinese and Western Medicine, Medical College of Nanchang University, Nanchang, 330006, China; ^3^ Department of Biotherapy, Sun Yat-sen University Cancer Center, Guangdong, 510060, China; ^4^ Department of General Surgery, Medical College of Nanchang University, Nanchang, 330006, China; ^5^ Department of Thoracic Surgery, Medical College of Nanchang University, Jiangxi Province Tumor Hospital, Nanchang, 330006, China; ^6^ Department of Integrated Chinese and Western Medicine, Jiangxi Province People's Hospital, Nanchang, 330006, China

**Keywords:** meta-analysis, crizotinib, anaplastic lymphoma kinase, non-small cell lung cancer

## Abstract

**Background:**

Crizotinib show a promising efficacy in patients with anaplastic lymphoma kinase (ALK)-positive non-small cell lung cancer (NSCLC). However, differences in efficacy for first- and second-line crizotinib are unclear.

**Results:**

The pooled overall response rate and progression-free survival were 65% and 9.38 months, respectively. In the subgroup analysis, first-line crizotinib showed a higher trend of overall response rate and longer trend of progression-free survival although there was no statistical difference between first-line and second-line crizotinib (74%, 11.28 months vs. 65%, 8.12 months, respectively; fixed effects model). Moreover, overall response rate between Asians and Caucasians were similar (67% and 66%, respectively; fixed effects model).

**Materials and Methods:**

A comprehensive search of MEDLINE, EMBASE, WEB OF SCIENCE and the COCHRANE databases from their inception to February 2016 was performed to identify clinical trials in English-language journals. Pooled overall response rate, progression-free survival and differences between first- and second-line crizotinib were estimated. Moreover, overall response rate between Asians and Caucasians were also estimated.

**Conclusions:**

First-line crizotinib may more effective than second-line crizotinib for patients with locally advanced or metastatic ALK-positive NSCLC.

## INTRODUCTION

According to the report of the World Health Organization, lung cancer is the leading incidence and the 5-year prevalence is about 36.5% in the world [1]. Non-small cell lung cancer (NSCLC) is the most common type of lung cancer and anaplastic lymphoma kinase (ALK) gene rearrangement has been demonstrated to be associated with approximately 2–7% of NSCLCs. ALK gene rearrangement is uncommon, however, it is more prevalent in younger patients who tend to be non-smokers or light smokers and who have adenocarcinomas [2, 3]. Crizotinib was approved by the United States Food and Drug Administration (FDA) in 2013 to treat patients with metastatic ALK-positive NSCLC and it is now approved in more than 85 countries [4]. The PROFILE 1001 study revealed that crizotinib achieved good efficacy and well tolerance in the treatment of ALK-positive NSCLC [5]. The PROFILE 1014 and PROFILE 1007 studies demonstrated that crizotinib was superior to chemotherapy in the first-line and the second-line settings, respectively [6, 7]; the efficacy and safety data from PROFILE 1029 study will be submitted for presentation at a future medical meeting [8]. Now new clinical trials, comparing next generation of ALK inhibitors with crizotinib, are ongoing (e.g. alectinib in the ALEX trial, ceritinib in the ASCEND-4 trial, brigatinib in the ALTA-1L trial).

A meta-analysis of clinical trials revealed that crizotinib was associated with a promising overall response rate (ORR: 61.2%, 95% confidence interval [CI]: 57.4–64.8%) and progression-free survival (PFS: 8.6 months [95% CI, 7.3–9.9 months]) in patients with locally advanced or metastatic ALK-positive NSCLC [9]. With the rapid development in oncology research and the increased number of clinical trials in recent years, updated data for crizotinib are available. However, the discrepancies in efficacy between first-line and second-line crizotinib are unclear. The objectives of this meta-analysis were evaluating the efficacy of crizotinib, assessing ORR between Asians and Caucasians, comparing the efficacy according to line of treatment in patients with locally advanced or metastatic ALK-positive NSCLC.

## RESULTS

### Primary characteristics of all studies

**Figure 1:**
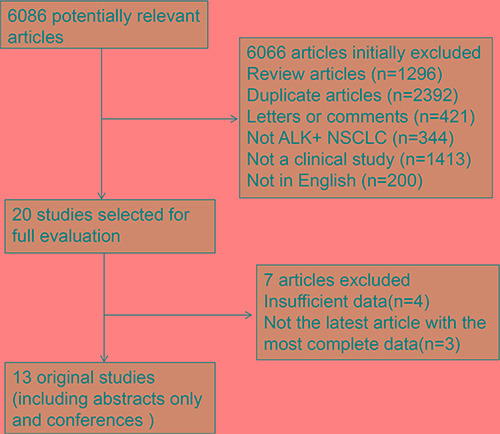
Flow diagram of the review ALK + NSCLC, anaplastic lymphoma kinase-positive non-small cell lung cancer

Our search yielded a total of 6086 articles. After evaluating each publication, we identified 13 original studies (representing 14 clinical trials; 1 study included 2 trials) [5–7, 10–19] that met our inclusion criteria. The details of our search results could be seen in the flow diagram (Figure 1). Among these 13 studies (including 11 non randomized control trials [NRCTs] and 2 randomized control trials [RCTs]), 3 studies described first-line therapies [7, 16, 17], 3 studies described second-line therapies [6, 11, 16] and 7 studies described mixed therapies [5, 10, 12–15, 19]. Moreover, 8 studies reported median PFS and 95% CI [5–7, 10, 13, 15, 16, 19]. The primary characteristics of the selected studies were listed in Table 1.

**Table 1:** Primary characteristics of the selected studies

Study	Study design	*N*	Median age	Female (%)	Never smoking (%)	Adeno- carcinoma (%)	Race (%)	Brain metastases (%)	Line of therapy (≥ Third, [%])	ECOG PS score ≤ 1 (%)
Camidge D Ross [5]	NRCTS	149	52	51	71	97	Caucasian 64 Asian 28 Other 9	0	Mixed-line (53%)	48
Kim, D. W [10]	NRCTS	255	53	53	65	92	NA	NA	Mixed-line (85%)	83
T. Asao [11]	NRCTS	6	54	50	NA	NA	Asian 100	NA	Second-line	67
M. Perol [12]	NRCTS	254	57	50	NA	NA	Caucasian 100	31	Mixed-line (53%)	76
Berta [14]	NRCTS	10	56	70	40	90	Caucasian 100	NA	Mixed-line (60%)	NA
Evelyn M. Brosnan [18]	NRCTS	38	54.7	47.4	NA	NA	Caucasian 94.7 Asian 0.02 Other 0.02	NA	NA	NA
CaoYabing [13]	NRCTS	40	42	42.5	NA	100	Asian 100	NA	Mixed-line (42.5%)	NA
Cui, Shaohua [15]	NRCTS	72	55	52.8	72.2	94.40	Asian 100	0	Mixed-line (25%)	97
Lei, Y. Y [19]	NRCTS	120	48	49.2	78.3	96.7	Asian 100	31.6	Mixed-line (NA)	93.7
Cui, S. a [16]	NRCTS	80	54	52	74	NA	Asian 100	0	First-line/second-line	97
Zhang, Q [17]	NRCTS	19	53	42.9	85.7	NA	Asian 100	NA	First-line	100
Alice T. Shaw [6]	RCTS	173	51	57	62	95	Caucasian 52 Asian 46 Other 2	35	Second-line	91
Benjamin J. Solomon [7]	RCTS	172	52	60	62	84	Caucasian 53 Asian 45 Other 2	26	First-line	94

a This study included two subgroups of trials: one underwent first-line treatment and the other underwent second-line treatment.

Abbreviations: NA, Not available; NRCTS, Non-randomized controlled trials; RCTS, Randomized controlled trials.

ECOG PS, Eastern Cooperative Oncology Group performance status.

### Study quality assessment and risk of bias

We summarized the methodological quality of all the NRCTs (excluding the abstracts only and conferences) in the Supplementary Table S1. The NOS results showed that the average overall score was 5.6 (range 5–7). None of the included studies had major flaws in assessment of their risk of bias. A common caveat, however, was the expected absence of blinded intervention. A detailed assessment of risk of bias was summarized in Supplementary Table S2.

### Pooled ORR of all studies

**Figure 2:**
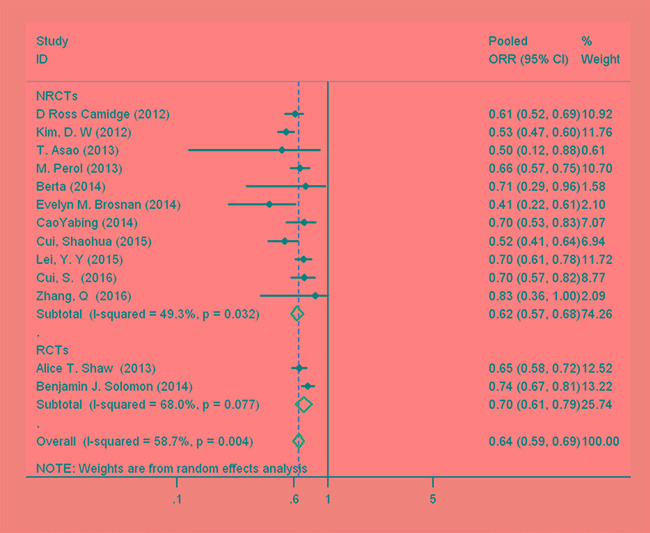
Forest plot showing the pooled overall response rate of patients with anaplastic lymphoma kinase (ALK)-positive non-small cell lung cancer (NSCLC) to crizotinib

Among the 13 studies [5–7, 10–19], the pooled ORR for crizotinib was investigated for treating ALK-positive NSCLC. An analysis of the pooled data revealed a pooled ORR (Figure 2) of 64% (95% CI, 59–69%, df = 12, *I*^2^ = 58.7%, *P* < 0.01).

**Figure 3:**
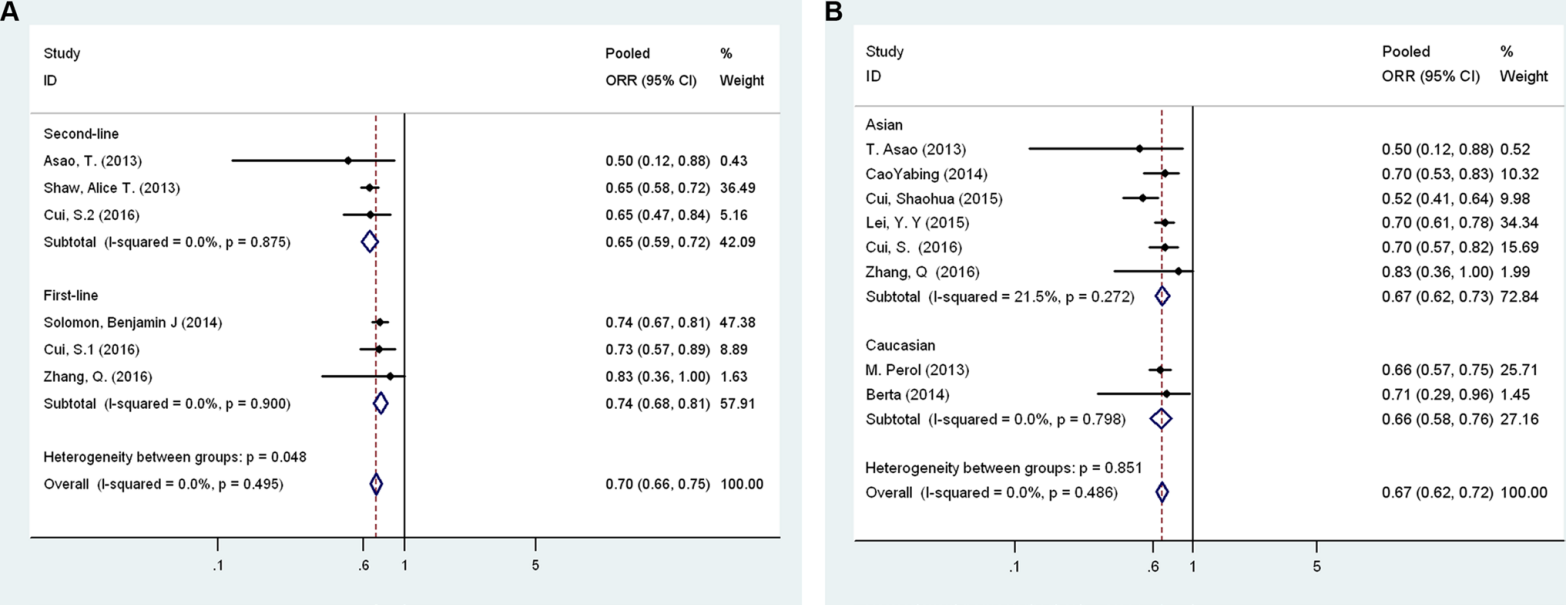
(**A**) Forest plot comparing the overall response rate to first-line and second-line therapy by patient subgroup. (**B**) Forest plot comparing the overall response rate for Asians and Caucasians by patient subgroup

For subgroups, a higher trend of first-line crizotinib for ORR could be seen although there was no statistical difference between first-line and second-line crizotinib. The ORR for first-line and second-line crizotinib were 74% (95% CI: 68–81%) and 65% (95% CI: 59–72%), respectively (Figure 3A). The pooled ORR of all patients who received crizotinib as either first- or second-line treatment was 70% (95% CI: 66–75%, *I*^2^ = 0.0%, *P = 0.495*) (Figure 3A). In the subgroup analysis of race, there were 8 studies that could be assessed [11–17, 19]. However, none statistically significant difference of ORR between Asians and Caucasians was detected (ORR, 67% [95% CI, 62–73%] and 66% [95% CI, 58–76%], respectively) (Figure 3B).

There was significant heterogeneity between studies in the pooled ORR analysis (*I*^2^ = 58.7%, *P* < 0.01). When stratified by study design, the heterogeneity was still significant for NRCTs and RCTs (Figure 2). From the results of the leave-one-out sensitivity analysis, all of the above results were not materially altered (data not shown). We found no evidence of publication bias in any analyses using Begg's (*P = 0.669*) and Egger's tests (*P = 0.481*).

### Pooled PFS of all studies

**Figure 4:**
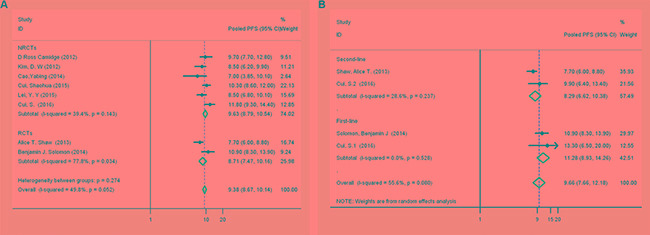
(**A**) Forest plot comparing progression-free survival to first-line and second-line therapy by patient subgroup. (**B**) Forest plot comparing progression-free survival by Asians and Caucasians by patient subgroup

There were 8 studies on the relationship of crizotinib use and PFS in ALK-positive NSCLC [5–7, 10, 13, 15, 16, 19]. An analysis of the pooled data revealed a pooled PFS (Figure 4A) of 9.38 months (95% CI, 8.67–10.14 months, df = 7, *I*^2^ = 49.8%, *P > 0.05*).

In the subgroup analysis between first-line and second-line crizotinib [6, 7, 16] (the study of Cui et al. [16] described 2 trials), the differences in PFS between first-line and second-line crizotinib treatment were not statistically significant (PFS, 11.28 months [95% CI, 8.93–14.26 months] and 8.29 months [95% CI, 6.62–10.38 months], respectively) (Figure 4B).

There was no significant heterogeneity between studies in the pooled PFS analysis (*I*^2^ = 49.8%, *P > 0.05*). From the results of the leave-one-out sensitivity analysis, all of the above results were not materially altered (data not shown). We also found no evidence of publication bias in any analyses using Begg's (*P = 1*) and Egger's tests (*P = 0.678).*

## DISCUSSION

We identified 6086 articles for review of title and abstract (Figure 1). After initial screening, we retrieved 20 potentially eligible articles for detailed data assessment. Four studies [20–23] were excluded with insufficient data: undescribed ORR and PFS. Three studies [3, 24, 25] from same trials were not the latest article with the most complete data and thus were excluded: one study [3] from PROFILE 1001 (ORR, 57%, 95% CI, 46–68%; median PFS was not reached) and two studies [24, 25] from PROFILE 1005 (one study didn't reported ORR and median PFS, another study reported ORR, 46 %, 95% CI, 42–50%; median PFS 8.1 months, 95% CI, 6.8–9.7 months). Therefore, the rest 13 articles were included in this meta-analysis.

In the present research, the ORR and PFS for crizotinib in the treatment of locally advanced or metastatic ALK-positive NSCLC were about 65% and 9.38 months, regardless of when crizotinib was used. Subgroup analysis between Asians and Caucasians showed the ORR of crizotinib was similar (67% vs. 66%, respectively). Therefore, crizotinib showed promising responses in both Asians and Caucasians with locally advanced or metastatic ALK-positive NSCLC. Moreover, the cumulative ORR and PFS for patients treated with first-line crizotinib were 74% and 11.28 months, respectively, in comparison to 65% and 8.12 months, respectively, for patients applied with second-line crizotinib. However, statistical significant difference was not detected between first-line and second-line crizotinib. Cui S *et al*. showed that the ORR was lower among those who received multiple-line (third-line or later) treatment prior to crizotinib therapy (*P = 0.702*) [15]. Thus, first-line crizotinib should be recommended as first-line treatment to patients with locally advanced or metastatic ALK-positive NSCLC [26]. Additionally, the National Comprehensive Cancer Network (NCCN) guideline (Version 1.2015) suggested crizotinib should be chose as first-line treatment for any performance status of ALK positive lung cancer [26]. Previous study indicated that crizotinib may enhance the efficacy of chemotherapeutic drugs especially for the patients with multidrug resistance in cancer chemotherapy [27]. However, for the patients with ALK mutation detected during first-line chemo-therapy, crizotinib should be utilized immediately or started after completing chemotherapy. For the patients with cancer progression after taking crizotinib, ceritinib should be started or keep taking crizotinib basing on whether the cancer causing symptoms or not [26].

Numerous clinical trials showed crizotinib treatment could prolong PFS and improve ORR [5–7]. Some novel findings have been found to be correlated with the response and PFS to crizotinib. Fluorescence *in situ* hybridization analysis demonstrated the percentage of ALK-positive cells was weakly correlated with the response to crizotinib [28]. Lei et al. showed that the ORR of crizotinib was similar between patients with and without brain metastases at baseline in Chinese patients with ALK-positive NSCLC (68.4% vs. 69.5%, *P = 0.904*) [19]. For patients with co-alterations of epidermal growth factor receptor (EGFR) mutations and ALK translocation, crizotinib also appeared to be effective [15, 29]. Many researches indicated the response rate seemed to be largely independent of age, sex, performance status, line of treatment, radical surgery history, histologic type and previous treatment received [5, 13, 15]. However, the patients with baseline good (0–2) performance status (PS) had a better PFS than those with poor (> 3) PS (50 weeks vs. 24 weeks, *P = 0.015*) [13]. Moreover, the patients without brain metastases at baseline have an extended median PFS (10.0 months, 95% CI, 7.6–12.5 vs. 7.0 months, 95% CI, 6.4–7.6; *P = 0.021*) [19]. Additionally, ALK-positive NSCLC patients with emergent central nervous system disease were more likely to benefit from treatment [30]. Although crizotinib showed good efficacy in most patients with ALK-positive NSCLC, patients invariably relapse typically within 1 year because of the intrinsic or acquired drug resistance [31]. Inhibitors of heat shock protein and second-generation ALK-inhibitor (ceritinib, alectinib, et al.) may have the potential to overcome the resistance to crizotinib [31–34].

Prospectively randomized clinical trials compared second-generation ALK inhibitors with crizotinib in the first-line setting are ongoing [35, 36]. However, single-arm trials showed that second-generation ALK inhibitor had clinical activity in patients with crizotinib-refractory or crizotinib-resistant ALK-positive NSCLC [37–39]. Moreover, J-ALEX study indicated that alectinib (second-generation ALK inhibitors) reduced the risk of disease worsening or death by 66 percent compared to crizotinib ((HR = 0.34, 99 percent CI: 0.17–0.70, *p < 0.0001*) and had the potential to become first-line therapy in patients with ALK-positive NSCLC [40]. Ceritinib (second-generation ALK inhibitors) showed better efficacy in ALK inhibitor-naive patients than ALK inhibitor-pretreated (all had received crizotinib, and five patients had also received alectinib) patients (ORR, 72% vs 56%; median PFS, 18.4 months vs 6.9 months) [37]. Similarly, alectinib (second-generation ALK inhibitors) showed longer PFS (the lowest PFS was 20.3 months) in the first-line treatment than second- and later-line treatment (the highest PFS was 11.3 months) [38, 40]. Consistent with the above results, present study indicated that crizotinib showed a trend of better efficacy in the first-line treatment than the second- and later-line treatment for patients with locally advanced or metastatic ALK-positive NSCLC; however, statistical significant difference was undetected.

Our meta-analysis has some important limitations. Although a sensitivity analysis was performed, a major limitation is the heterogeneity that existed among the results of all included studies. Moreover, this was a meta-analysis of summary estimates rather than an analysis of individual patient data, which could have provided further insight into the efficacy of the crizotinib treatment. Therefore, the results should be interpreted prudently; compared to an individual patient database systematic review based on abstracted data or published data that would lack reliable evaluation. Additionally, publication bias might have occurred because we did not include unpublished data, although evidence of publication bias was not found based on results of the Begg's test and Egger's test. Finally, the number of studies included that used first-line and second-line therapy was small, so statistical power was limited.

In conclusion, the meta-analysis estimated the pooled ORR and PFS of crizotinib in patients with locally advanced or metastatic ALK-positive NSCLC and compared the efficacy of first-line and second-line treatment. Our analysis demonstrated that among patients with locally advanced or metastatic ALK-positive NSCLC, crizotinib showed effective response rate and appears to be a favourable treatment option. Moreover, first-line crizotinib may more effective in comparison with second-line crizotinib for patients with locally advanced or metastatic ALK-positive NSCLC. However, further multicentre, RCTs with larger sample sizes are needed to compare the efficacy of first-line and second-line therapy.

## MATERIALS AND METHODS

### Search strategy for identifying studies

A comprehensive search of the MEDLINE, EMBASE, WEB OF SCIENCE, and COCHRANE databases from their inception to February 2016 was performed to identify clinical trials in English-language journals. The following medical subject heading (MeSH) terms and text words were used in combination: “pulmonary” or “lung”, “cancer’’ or ‘‘tumour’’ or ‘‘carcinoma’’, ‘‘ALK’’ or ‘‘anaplastic lymphoma kinase’’, ‘‘crizotinib’’.

According to the PICO checklist, eligibility criteria were as follows: (1) population: patients with locally advanced or metastatic anaplastic lymphoma kinase-positive non-small cell lung cancer; (2) intervention: crizotinib; (3) control: none; (4) outcome: ORR (defined as the observed survival rate of patients since the date of crizotinib treatment) and PFS (time from beginning of treatment to non-small cell lung cancer recurrence or death).

We also manually searched the reference lists of all pertinent studies. Meeting abstracts from the American Society of Clinical Oncology, the European Society for Medical Oncology and ClinicalTrials.gov were also hand-searched to identify eligible trials. Finally, reference lists of original articles and review articles were also searched. The corresponding authors of some studies were contacted for further information if necessary. Our analyses were conducted in accordance with the Preferred Reporting Items for Systematic Reviews and Meta-analyses (PRISMA) guidelines (when appropriate) for a systematic review of prevalence [41].

### Study selection

All clinical trials that explored the efficacy of crizotinib for the treatment of locally advanced or metastatic ALK-positive NSCLC were considered eligible for analysis. The inclusion criteria were as follows: (1) articles were clinical trials investigating the efficacy of crizotinib for the treatment of patients with locally advanced or metastatic ALK-positive NSCLC; (2) a standardized effect size could be calculated on the evaluations of ORR and PFS; (3) articles were in English and the dose and frequency of crizotinib administration was 250 mg twice daily, which was approved by the FDA; (4) the included study must have sufficient data available for extraction; (5) when multiple articles were based on the same trial, only the latest article with the most complete data was included for this meta-analysis. Two investigators (X. Yang and H. Wang) independently assessed the articles for relevance to our study.

### Study quality assessment

The full texts of non-randomized clinical trials were assessed using the 9-point Newcastle Ottawa scale (NOS) by two investigators (H.D. Wang and Q. Zhu). Two investigators independently evaluated each study based on eight items, categorized into three broad perspectives including selection, comparability and outcome for cohort studies or exposure for case-control studies [42]. We considered studies with a score of 7 or greater as high quality. Risk of bias in the included studies was independently assessed by two investigators using the Cochrane collaboration's tool for assessing risk of bias in randomized control trials (RCTs) [43]. Each study was independently assessed by two authors (H.D. Wang and Q. Zhu) under five main headings for risk of bias. Disagreements were resolved by discussion or through consultation with the senior reviewer.

### Data extraction

According to the inclusion and exclusion criteria, two of our investigators independently completed the retrieval process. For each article, we collected the first author, year of publication, number of patients enrolled, number of patients analysed, therapeutic regimen, demographic factors (such as age and histologic type), ORR and PFS for patients. We collected the data of each line of clinical trials through the mentioned related information about each line in section of inclusion criteria or method, respectively. “Patients received no previous systemic treatment (ie, chemotherapy or others)” were defined as first line therapy. “Patients receive crizotinib after one prior platinum-based chemotherapy regimen” were defined as second line therapy. “Patients receive crizotinib first-line and/or some patients with second-line and/or others receive crizotinib more than one prior platinum-based chemotherapy regimen” were defined as mixed line therapy. The abstracts of the articles were independently reviewed by two authors (H. Hu and W. Q. Lin). Outcomes were pooled to obtain the ORR and PFS. Data were filtered and transferred into a standard electronic form. Any discrepancies were resolved by discussion until a consensus was reached. If an agreement was not reached, the principal investigator (Y. K. Kuang) made the final decision on the eligibility of the study and data extraction.

### Statistical analysis

Stata Statistical Software, version 13.0 (Stata Corporation, College Station, TX, USA) was used to analyse the extracted data. Data were pooled statistically using the event rates calculated for ORR. The heterogeneity of the estimators was assessed with a χ^2^ test and the I^2^ statistic, which describes the percentage of the variability in effect estimates caused by heterogeneity [44]. Summary ORR and PFS estimates were based on random-effect models where *I*^2^ ≥ 50% and fixed models for *I*^2^ < 50%. We considered that heterogeneity was present when the Cochran's *Q*-test *P*-value was < 0.05. When results of two heterogeneity statistics are discrepant, to be more conservative, we considered that heterogeneity was present and random effects model was applied. All tests were 2-sided and statistical significance was defined as *p* < 0.05. Finally, both the Begg's test and Egger's test were used to estimate potential publication bias [45, 46]. A sensitivity analysis was used to verify value stability.

## SUPPLEMENTARY MATERIALS



## Abbreviations

ALK: anaplastic lymphoma kinase
CI: confidence interval
FDA: Food and Drug Administration
MeSH: medical subject heading
NOS: Newcastle Ottoman scale
NRCT: non-randomized controlled trial
RCT: randomized controlled trial
NSCLC: non-small cell lung cancer
ORR: overall response rate
PFS: progression-free survival
PS: performance status
EGFR: epidermal growth factor receptor
NCCN: National Comprehensive Cancer Network
