# Supplementary material for: Is there a benefit of first- or second-line crizotinib in locally advanced or metastatic anaplastic lymphoma kinase-positive non-small cell lung cancer? a meta-analysis
Source: Oncotarget. 2016 Nov 7;7(49):81090–8. doi: 10.18632/oncotarget.13191 (PMC5348378; doi:10.18632/oncotarget.13191)
Supplement: Supplementary file 1 [file oncotarget-07-81090-s001.pdf]

Is there a benefit of first- or second-line crizotinib in locally advanced or metastatic anaplastic lymphoma kinase-positive non-small cell lung cancer? a meta-analysis

Supplementary Materials

Supplementary Table S1: Nine-point newcastle ottawa scale scores for the non-randomized controlled trials

Domain	Item	Camidge D [5]	Brosnan,E. M. [18]	Yabing Cao [13]	Cui,Shaohua [15]	Lei,Y.Y [19]	Cui,S. [16]	Quan Zhang [17]
Selection	(Maximum of four stars)							
	Representativeness of the exposed cohort							
	Selection of the non-exposed cohort							
	Ascertainment of exposure	*	*	*	*	*	*	*
	Demonstration that outcome of interest was not present at start of study	*	*	*	*	*	*	*
Comparability	(Maximum of two stars)							
	Comparability of cohorts on the basis of thedesign or analysis						**	**
Outcome	(Maximum of three stars)							
	Assessment of outcome	*	*	*	*	*	*	*
	Was follow-up long enough for outcometo occur	*	*	*	*	*	*	*
	Adequacy of follow-up of cohorts	*	*	*	*	*	*	*

A study can be awarded a maximum number of four stars for the selection domain, maximum of two stars for the comparability domain and maximum of three stars for the outcome domain.

Supplementary Table S2: Risk of bias in randomized controlled trials

Study	Selection bias	Performance bias	Detection bias	Attrition bias	Reporting bias	Other bias
Shaw, Alice T.[6]	Unclear – no description of random sequence generation or allocation concealment	High – no blinding of participants or personnel	High – no blinding of participants or personnel	Low – all trial participants were accounted for	Low – no selective reporting	Low
Benjamin J. Solomon [7]	Unclear – no description of random sequence generation or allocation concealment	High – no blinding of participants or personnel	High – no blinding of participants or personnel	Low – all trial participants were accounted for	Low – no selective reporting	Low
